# The Impact of Relationships Within Combat Units on Post-Deployment Suicide Risk

**DOI:** 10.3390/bs14111040

**Published:** 2024-11-05

**Authors:** Leo Sher

**Affiliations:** 1 Inpatient Psychiatry, James J. Peters VA Medical Center, New York, NY 10468, USA; leo.sher@mssm.edu; 2 Department of Psychiatry, Icahn School of Medicine at Mount Sinai, New York, NY 10029, USA; 3 Department of Psychiatry, Columbia University Vagelos College of Physicians and Surgeons, New York, NY 10032, USA

**Keywords:** combat veteran, deployment, unit cohesion, harassment, suicide

## Abstract

Multiple deployment factors may affect suicidality in combat veterans. The relationships between combat deployments and suicidality are complex and not completely understood. Studies of stress in the military and psychological effects of military actions are mostly focused on stressors relating to combat operations. However, many studies suggest that interactions within combat units affect post-deployment psychiatric conditions, suicidal ideation, and behavior. The goal of this article is to review and discuss how relationships within combat units may influence post-deployment suicide risk. Studies of the relationships within combat units are generally focused on two aspects: unit cohesion and harassment/abuse. Considerable evidence suggests that service members who report strong unit cohesion have a lower risk of post-deployment psychiatric disorders and suicidal behavior. Studies examining deployment sexual and non-sexual harassment and abuse have found that combat veterans who experience harassment and abuse during deployment are at heightened post-deployment suicide risk. Sound post-deployment social support and the efficient treatment of psychiatric disorders may mitigate the suicide risk associated with adverse relationships within combat units. Improvements in units’ cohesion and the prevention of harassment/abuse during a military deployment are necessary to reduce post-deployment psychiatric pathology, including suicidal behavior.

## 1. Introduction

Wars have been a part of the history of humankind. Regretfully, there is no evidence that a time free of war will become a reality in the foreseeable future. According to a recently published report, “Global Peace Index 2024: Measuring Peace in a Complex World” there are 56 military conflicts around world at the present time [1]. This is the largest number of military conflicts since the Second World War. According to this report, 92 countries are engaged in military actions beyond their borders. The number of minor military conflicts is increasing. This raises the possibility that there will be more major wars in the future.

There are millions of combat veterans around the globe. For example, after the terrorist attacks of 11 September 2001, between 1.9 and 3 million members of the U.S. Armed Forces served in military operations in Iraq and Afghanistan and over half of them were deployed more than once [2]. It should be noted that in the U.S., a military veteran is a person who served in the Armed Forces [3]. In the U.S., combat veterans are those who served in an area of conflict/war while in the Armed Forces. In many countries, individuals are regarded as military veterans if they served during wartime and/or participated in combat operations; i.e., only combat veterans are classified as veterans [4].

Combat conditions are characterized by violence, brutality, physical and emotional strain, separation from families and friends, and other adversities. Major physical combat injuries are associated with psychiatric disorders including post-traumatic stress disorder (PTSD), depression, anxiety, and suicidality [5,6,7]. For example, a study of the UK military servicemembers who were deployed to Afghanistan showed that servicemembers who had physical combat injuries were 46–67% more likely to report PTSD, depression, and anxiety in comparison with uninjured servicemembers [7].

Multiple deployment factors may affect suicidality in combat veterans [8]. The relationships between combat deployments and suicidality are complex and not completely understood. Studies of stress in the military and the psychological effects of military actions are mostly focused on stressors relating to combat operations, such as traumatic exposure to death and injuries, exhaustion, and being exposed to extreme climates [9]. However, many studies performed over the last several decades suggest that interactions within combat units affect post-deployment psychiatric conditions and suicidal ideation and behavior [10,11,12,13,14,15,16,17,18,19,20,21]. These studies are generally focused on two aspects: unit cohesion and harassment/abuse (See Table 1) The goal of this article is to review and to discuss how relationships within combat units may influence post-deployment suicide risk.

## 2. Suicidality in Combat Veterans

Suicide among combat and non-combat veterans is a critical public health issue that causes incalculable pain among individuals, families, and communities [8,23,24]. According to the 2022 VA National Veteran Suicide Prevention Annual Report published by the Office of Mental Health and Suicide Prevention of the U.S. Department of Veterans Affairs, there were 6146 veteran suicide deaths in the U.S. in 2020 [24]. Military veterans have higher rates of suicide than individuals in the general population. According to the above-mentioned report, the suicide rate for veterans was 57.3% higher than non-veteran U.S. adults in 2020, after adjusting for age and sex differences [24]. An epidemiological study performed by psychiatric researchers in Michigan found that among veterans from different periods of military service treated in the U.S. Veterans Health Administration (VHA) medical facilities, male veterans were 1.66 times more likely to die by suicide than males in the general population, and female veterans were 1.87 times more likely to die by suicide than females in the general population [23].

Studies of the U.S. veterans of Operation Enduring Freedom (OEF), Operation Iraqi Freedom (OIF), and Operation New Dawn (OND), and other U.S. and international veterans, suggest that many combat veterans exhibit suicidal ideation and behavior [8,10,25,26]. A survey of 272 OEF/OIF veterans in Connecticut found that 12.5 percent of study participants had contemplated suicide in the 2 weeks preceding the study [10]. The Army Study to Assess Risk and Resilience in Servicemembers performed by a large group of U.S. researchers found a higher suicide rate in service members who were currently deployed or had been previously deployed compared with those who had never been deployed [27].

A more recent study of the risk of suicide among U.S. combat veterans found that there was an increased risk of suicide when all OEF/OIF/OND veterans were compared with the U.S. general population [28]. Researchers from the U.S. Department of Veterans Affairs used records from the U.S. Department of Defense to identify the study cohort. They found that both male and female veterans had an increased risk of suicide when compared to their gender-specific non-veteran counterparts. About 70 percent of veterans who died by suicide killed themselves using a firearm. After 15 years of follow-up, OEF/OIF/OND veterans continued to have a suicide rate that exceeded a suicide rate in the U.S. general population. A retrospective multivariate analysis of all U.S. military personnel between 2001 and 2011 that included about 3.8 million service members found that OEF and OIF veterans had elevated suicide rates for the first 4 years after their deployment in comparison with military personnel who had not been deployed [29]. This indicates that the risk of suicide is the highest during the first years after a war-zone deployment. Although some studies did not find relationships between suicide and deployments to OEF or OIF, some subgroups of combat veterans appeared to be more vulnerable to suicide than other combat veterans [30,31].

In 2002, U.K. veterans of the Falklands War (April-June 1982) claimed that more Falklands veterans had died by suicide (N = 264) since the conflict ended than died during the conflict itself (N = 256) [32]. Suicide rates among U.K. veterans of the 1991 Gulf War were also significant [32].

## 3. Unit Cohesion

Unit cohesion refers to the ties that preserve service members’ dedication to each other, the unit, and the mission [33]. The concept of unit cohesion includes trust, reliance, and comradeship among peers (horizontal cohesion), as well as support, help and encouragement from, and respect for, unit leadership (vertical cohesion) [33,34]. Vertical cohesion can be described as leader-led support. Unit cohesion in the Armed Forces is critically integral to individual and group well-being, functioning, and security. Military unit cohesion strongly affects psychological and physical outcomes [12,35,36,37].

One of the important experiences of the Second World War was the identification of the critical significance of team cohesion in preventing psychiatric issues among the soldiers [35,36]. It has been suggested that “WWII clearly showed that interpersonal relationships and other social and situational circumstances were at least as important as personality configuration or individual assets and liabilities in the effectiveness of coping behavior” [35].

Considerable evidence suggests that servicemembers who report strong unit cohesion have a lower risk of psychiatric disorders and suicidal ideation [10,12,15,17,19]. A study showed that among U.S. Army soldiers, a higher perceived unit cohesion approximately 1–2 months before deployment to Afghanistan was associated with a lower risk of PTSD, major depression, generalized anxiety disorder, substance use disorder, and suicidal ideation 3 and 9 months after return from deployment [19]. In one study mentioned above [10], the authors used the Unit Support Scale (USS), a 12-item self-report instrument from the Deployment Risk and Resilience Inventory (DRRI) [34,38] that assesses the amount of assistance and encouragement in the war-zone from unit leaders and members and the military in general. The authors found that veterans who reported post-deployment suicidal ideation had lower scores on measures of unit support [10].

Griffith [17] from the U.S. National Center for Veterans Studies examined relationships among combat exposure, post-deployment problems, social help, and unit cohesion. In all, 4567 formerly deployed U.S. servicemembers who were soldiers of 50 company-sized units were surveyed. The study showed that at the group level, a diminished risk for suicidal ideation was associated with units having better than typical cohesion [17]. A study of 1663 U.S. servicemembers performed by a research group from the U.S. Army Institute of Public Health showed that combat exposure was a significant risk factor for suicidal ideation, while unit cohesion was a significant protective factor [13]. The significant interaction between the two factors indicated that soldiers who experienced greater combat exposure but also had higher levels of unit cohesion had relatively lower levels of suicide-related ideation. In addition, those who had higher levels of combat exposure and lower unit cohesion were most at risk for suicide-related ideation.

A study of risk and protective factors for suicidal ideation and suicide attempts among deployed Danish soldiers found that a heavy workload was associated with suicidal ideation and pointless tasks were a predictor for suicide attempts [16]. Pointless tasks can include repetitive chores when no military duties are available or military operations that are illogical from the soldiers’ point of view. The perception or presence of heavy workloads and/or pointless tasks may be related to weak vertical cohesion.

## 4. Sexual and Non-Sexual Harassment and Abuse

Studies examining deployment sexual and non-sexual harassment and abuse have found that combat veterans who experience harassment and abuse are at heightened suicide risk [11,14,18,20,21]. This is consistent with observations among civilians indicating that different types of harassment and abuse lead to mental health issues and suicidality [39,40,41,42]. This underlines the importance of the prevention of harassment and abuse in different groups of the population.

A cross-sectional retrospective analysis of psychological evaluations of 1740 OEF/OIF veterans performed by researchers in Texas showed that general (non-sexual) harassment was associated with post-deployment suicidal ideation [11]. We performed a study on the relation of harassment during a combat deployment with post-deployment suicidality and testosterone function in male veterans [21]. We found that harassment scores were higher, and free testosterone levels were lower, among veterans who made post-deployment suicide attempts in comparison with veteran non-attempters. In the whole sample, harassment scores positively correlated with suicidal ideation scores (r = 0.40, *p* = 0.02) and negatively correlated with free testosterone levels (r = −0.36, *p* = 0.04). Free testosterone levels negatively correlated with suicidal ideation scores (r = −0.57, *p* < 0.001). Our observations may indicate that there is a link between deployment harassment, testosterone function, and suicidality. Our study is consistent with previous observations indicating that suicidal behavior in combat veterans has a neurobiological basis [8,22,43]. 

Kimerling et al. [18] examined data on veterans who received VHA services in the fiscal years 2007–2011 and were screened for military sexual trauma (MST; 5,991,080 men; 360,774 women). In the VHA, the term MST is used to refer to sexual assault or repeated, threatening sexual harassment during military service [44]. MST was reported by 1.1% of men and 21.2% of women. A total of 9017 veterans died by suicide during the follow-up period. Hazard ratios for military sexual trauma were 1.69 (95% CI = 1.45, 1.97) among men and 2.27 (95% CI = 1.76, 2.94) among women. Another study found that among female veterans, sexual harassment was the only deployment stressor to have an independent association with suicidal ideation, even when accounting for depression and alcohol-use symptoms [14]. Sexual harassment occurring during deployment may have an especially harmful effect on female veterans above and beyond its impact on psychiatric symptoms. A more recent study of women veterans who use VHA reproductive health care services performed by researchers in Colorado showed that the elevated prevalence of suicidal ideation after military service was associated with both military sexual harassment and assault [20]. Post-military suicide attempts were associated with military sexual assault. Studies by Kimerling et al. [18] and Monteith et al. [20] included both combat and non-combat veterans.

## 5. Post-Deployment Interventions to Decrease Suicidality

Sound post-deployment social support and the efficient treatment of psychiatric disorders may mitigate the suicide risk associated with adverse relationships within combat units [10,45,46,47]. A study on a longitudinal cohort derived from the Survey of Experiences of Returning Veterans that included 824 (480 men, 344 women) previously deployed OEF/OIF/OND veterans showed that among men, the association between deployment sexual trauma and suicidal ideation was moderated by post-deployment social support [47]. The authors also found that deployment sexual trauma predicted suicidal ideation only for men reporting that their post-deployment social support was low. Among women, post-deployment support mediated the association between deployment sexual trauma and suicidal ideation. The inclusion of post-deployment support in the model decreased the magnitude of the association between deployment sexual trauma and suicidal ideation. Another study showed that increased post-deployment social support and feelings of purpose and control were associated with fewer suicidal thoughts [10]. 

A study of 145 OEF/OIF veterans revealed that symptoms of PTSD and depression had almost no effect on suicidal ideation when post-deployment social support was high; however, when post-deployment social support was low, symptoms of PTSD and depression were positively associated with suicidal ideation [46]. Therefore, it is crucial to help veterans who have suffered deployment harassment and/or abuse to obtain social support after returning from deployment. A sense of purpose is frequently essential to recovery for these veterans. Also, for veterans reporting poor perceived post-deployment support, evaluating suicidality may be especially important. It is also very important to provide psychoeducation for the families and friends of returning veterans to emphasize the significance of post-deployment support and to educate them on how to recognize the warning signs of suicide [48,49]. Families are vital to recovery and are often the first to recognize suicidality in veterans. 

The effective psychological and pharmacological treatment of psychiatric symptoms is a useful point of intervention for veterans experiencing suicidality after deployment. Psychotherapeutic interventions including cognitive–behavioral therapy and supportive therapy may reduce symptoms of depression, PTSD, and other psychiatric disorders and, consequently, reduce suicidality [50,51].

Antidepressants and other medications may reduce symptoms of depression, anxiety, PTSD, and other psychiatric disorders and, therefore, reduce the suicide risk in veterans [45,50,51]. It is important to note that even mild symptoms of depression and PTSD may increase the risk of suicide. The continuity of care is critical when dealing with veterans who have suicidal ideation or a history of attempted suicide. Such veterans need an effective outpatient follow-up upon discharge from the emergency department or inpatient care.

## 6. Organizational Changes

As described above, considerable evidence suggests that relations within combat units influence the development of post-deployment psychiatric illnesses, including suicidal ideation and behavior. More specifically, it has been shown that poor unit cohesion and the presence of harassment and/or abuse may have a deleterious effect on the mental health of combat veterans. Therefore, improvement in units’ cohesion and the prevention of harassment/abuse during a military deployment are necessary to reduce post-deployment psychiatric pathology, including suicidal behavior [8,9,11,12,35,36,37]. This should be a priority in the military. 

Commanding officers can prevent harassing behavior, because they monitor and manage military servicemembers [17,52]. In 2018, the U.S. Department of Defense established a policy on harassment, “Harassment Prevention and Response in the Armed Forces” [52]. This policy comprises all types of harassment, including general harassment, “discriminatory” harassment (based on race, color, religion, sex, etc.), sexual harassment, bullying, hazing, retaliation, and reprisals. According to this policy, military leadership is expected to take appropriate disciplinary or administrative actions against the harasser if harassment complaints are confirmed.

Effective leaders that recognize psychiatric problems among service members and address mental health stigma, collaborate with mental health professionals, and support ethical and spiritual practices may reduce suicidal ideation and behavior among military personnel [53,54]. Some service members may have had an undiagnosed psychiatric disorder upon entry into the Armed Forces, and the distress related to that disorder only became evident during their time in military service. Some service members may have also had a predisposition to a psychiatric condition that developed during military service because of the stress. For example, the military environment can serve as a facilitator for the development and progression of depression [54]. The actions of military leaders toward servicemembers with mental health problems and communication strategies surrounding suicidal behavior may significantly influence the psychological condition of affected soldiers. It is important to support public health interventions, with a view to fostering screening, enhanced access, and greater coordination with unit leaders. Also, it is essential that military personnel are educated about the importance of a non-judgmental and supportive attitude toward their peers with mental health issues.

Sexual minority service members encounter distinct stressors and issues which may affect the mental health of this demographic group. For example, a cross-sectional survey by McDonald et al. [55] showed that a higher proportion of lesbian, gay, or bisexual soldiers than heterosexual soldiers screened positive for anxiety, PTSD, and suicidality. The mental health of sexual-minority individuals in the military represents a crucial area of investigation.

Military commanders influence service members and activities that make up the Armed Forces. The diligent selection and effective training of military leaders are of utmost importance. Effective military leaders are expected to value their subordinates, protect them from harassment, and efficiently work to improve vertical and horizontal cohesion [9,17,52,56]. Trustworthiness, credibility, skills, knowledge, concern for morale, instilling a sense of purpose, and providing regular feedback are vital to successful military leadership.

The studies referenced in this review cover a wide chronological span. It is important to note that conditions in the military may change over time, especially considering that weapons of war are becoming more powerful, sophisticated, accurate, and destructive.

## 7. Conclusions

Weak unit cohesion and the existence of harassment and/or abuse have a harmful impact on the psychological health of servicemembers and veterans. Moreover, such adverse circumstances may negatively affect the ability of combat units to perform military tasks. Therefore, efforts should be made to reduce harmful relations within combat teams. Mental and non-mental health medical practitioners need to be educated in the importance of identifying servicemembers and veterans who are victims of adverse relations within military units and proving them with appropriate psychiatric and medical treatment. Military leaders and policy makers should try to improve team cohesion and to reduce harassment/abuse in combat units. The prevention of mental health issues including suicidal behavior in military personnel and veterans is of utmost importance. 

## Figures and Tables

**Table 1:** Studies on the relations between suicidality and combat unit cohesion and between suicidality and deployment harassment/abuse.

Authors	Purpose	Study Design	Sample	Main Findings
Anderson et al. [19]	To evaluate prospective associations of perceived unit cohesion with mental health outcomes following combat deployment	A survey before and after deployment to Afghanistan	4645 soldiers	Soldiers who reported strong unit cohesion before deployment had a lower risk of post-deployment mental disorders and suicidal ideation
Bryan and Heron [15]	To study factors that protect against depression in military servicemembers	Self-report measures of depression, posttraumatic stress, and sense of “belonging” before and after deploying to Iraq	168 Air Force convoy operators	A sense of belonging may protect service members from depression at all stages of the deployment cycle
Du Preez et al. [12]	To examine the association between unit cohesion and probability of PTSD or other mental disorders	Information was collected on socio-demographic and military characteristics and deployment experiences	4901 male UK armed forces personnel deployed to Iraq	Unit cohesion had a linear association with less probable PTSD and common mental disorder
Ejdesgaard et al. [16]	To identify risk and protective factors for suicidal ideation and suicide attempts among deployed Danish soldiers	A questionnaire survey	1264 Danish soldiers deployed from 1990 to 2009	A heavy workload was associated with suicidal ideation. Pointless tasks were a predictor for suicide attempts
Gradus et al. [14]	To examine the role of post-deployment mental health in associations between deployment stressors and post-deployment suicidal ideation	A questionnaire survey	A national sample of 2321 female and male OEF/OIF veterans	Among female veterans, sexual harassment was the only deployment stressor to have an independent association with suicidal ideation
Griffith [17]	To examine relationships among combat exposure, post-deployment stressors, social support, and unit cohesion	A questionnaire survey	4567 combat soldiers who were members of 50 company-sized units	At the group level, a diminished risk for suicidal ideation was associated with units having better than typical cohesion
Kimerling et al. [18]	To assess associations between military sexual trauma screen results and subsequent suicide mortality	Screening for military sexual trauma	Veterans who received Veterans Health Administration services in the fiscal years 2007–2011: 5,991,080 men and 360,774 women	A total of 9017 veterans died by suicide during the follow-up period. The hazard ratios for military sexual trauma were 1.69 (95% CI = 1.45, 1.97) among men and 2.27 (95% CI = 1.76, 2.94) among women
Kovacic et al. [22]	To examine platelet serotonin concentration in male combat veterans and non-veterans	A survey and determination of platelet serotonin concentration	73 suicidal and 47 non-suicidal veterans with current and chronic combat-related PTSD, 45 suicidal and 30 non-suicidal comparative non-PTSD subjects, and 147 healthy men	A reduced platelet serotonin concentration is related to suicidal behavior in PTSD
Lemaire and Graham [11]	To examine factors associated with suicidal ideation in returning Iraq and Afghanistan war veterans	A cross-sectional review of veterans’ initial mental health screening evaluations	1740 OEF/OIF veterans	General (non-sexual) harassment was associated with post-deployment suicidal ideation
Mitchell et al. [13]	To test the associations between combat exposure, unit cohesion, and their interaction in predicting suicide-related ideation	A survey using rating scales	1663 recently redeployed soldiers	Soldiers who experienced greater combat exposure but also had higher levels of unit cohesion had relatively lower levels of suicide-related ideation. Soldiers who had higher levels of combat exposure and lower unit cohesion were most at risk for suicide-related ideation
Monteith et al. [20]	To evaluate whether military sexual trauma is associated with post-military suicidal ideation and suicide attempt history	A secondary analysis of quantitative data collected from a survey	2250 post-9/11 female veterans	An elevated prevalence of suicidal ideation after military service was associated with both military sexual harassment and assault
Pietrzak et al. [10]	To examine variables associated with suicidality in OEF/OIF veterans	A questionnaire survey	272 OEF/OIF veterans	Veterans who reported post-deployment suicidal ideation had lower scores on measures of unit support
Sher et al. [21]	To test the hypothesis that harassment during a combat deployment is associated with post-deployment suicidality and testosterone function	An interview using rating scales	32 male combat veterans with or without a history of suicide attempts	Harassment scores were higher and free testosterone levels were lower among suicide attempters in comparison with non-attempters

## Data Availability

Not applicable.
